# Menopause depression: Under recognised and poorly treated

**DOI:** 10.1177/00048674241253944

**Published:** 2024-05-18

**Authors:** Jayashri Kulkarni, Caroline Gurvich, Eveline Mu, Grace Molloy, Sonya Lovell, Ginni Mansberg, Shelly Horton, Erin Morton, Talat Uppal, Ceri Cashell, Anthony de Castella, Dan Reisel, Linda Dear, Naomi Weatherburn-Reeves, Katie Harris, Kerry Pietrobon, Kelly Teagle, Bo Youn Kim, Louise Newson, Cassandra Szoeke

**Affiliations:** 1 HER Centre Australia, School of Translational Medicine, Monash University, Melbourne, VIC, Australia; 2 Menopause Friendly Australia, Surry Hills, NSW, Australia; 3 Dear Menopause Australia, Sydney, NSW, Australia; 4 Don’t Sweat It Australia, Sydney, NSW, Australia; 5 Health Data and Clinical Trials, Flinders University, Adelaide, SA, Australia; 6 Women’s Health Road, Frenchs Forest, NSW, Australia; 7 Avalon Family Medical Practice, Avalon Beach, NSW, Australia; 8 Newson Health Ltd, London, UK; 9 Menodoctor, Tauranga, New Zealand; 10 Lived Experience & Menopause Advocate , Brunswick, VIC, Australia; 11 Zebra Research Pty Ltd, Sydney, NSW, Australia; 12 WellFemme, Canberra, ACT, Australia; 13 WA Country Health Service, Perth, WA, Australia; 14 Healthy Ageing Program, Faculty of Medicine, Dentistry and Health Sciences, The University of Melbourne, Parkville, VIC, Australia

**Keywords:** Menopause, gonadal hormones, menopause hormone therapy

## Abstract

Menopause is a biological process experienced by all people assigned female at birth. A significant number of women experience mental ill health related to the major brain gonadal hormone shifts that occur in their midlife. There is poor understanding and management of the complex mental ill health issues, with the biological brain hormone changes receiving little formal attention. The current treatment advice is to manage this special type of mental ill health in the same way that all mental ill health is managed. This leads to poor outcomes for women and their families. Many women leave the workforce earlier than expected due to menopause-related depression and anxiety, with subsequent loss of salary and superannuation. Others describe being unable to adequately parent or maintain meaningful relationships – all ending in a poor quality of life. We are a large and diverse group of national and international clinicians, lived experience and social community advocates, all working together to innovate the current approaches available for women with menopausal mental ill health. Above all, true innovation is only possible when the woman with lived experience of menopause is front and centre of this debate.

On 6 November 2023, the Australian Senate referred an inquiry into issues related to menopause and perimenopause to the Senate Community Affairs References Committee for inquiry and report by 10 September 2024. No doubt many submissions will be made from private citizens and diverse groups in our community. The anticipated issues to be raised will include physical health, financial issues including loss of earnings and superannuation through premature retirement and the provision of special health services.

In this article, we focus on the rarely discussed and seldom recognised mental health issues caused by the menopausal process.

Middle-aged women, compared to other age groups of women, experience significant increases in depression, anxiety, post-traumatic stress disorder and substance abuse. As a demographic cohort, middle-aged women have a high suicide rate (Blazer et al., 1994; Cohen et al., 2006; Dennerstein et al., 2004; Maki et al., 2019; Tangen and Mykletun, 2008). Yet this group’s specific needs remain somewhat invisible and unmet. For decades, the global rise in midlife mental ill health has been considered to be a product of the complexity of women’s lives. Social determinant theories about midlife depression in women detail the cause as stresses experienced in many domains of life such as in paid and unpaid workplaces, in intimate relationships, when raising children and caring for sick or elderly family members, while maintaining social networks and the stress of being concerned with ageing in a youth-oriented society (Austen and Viforj, 2010; Cohen et al., 2006; Pimenta et al., 2012).

No doubt many middle-aged women juggle all these stresses – but the social determinant theories do not explain the sudden onset of severe anxiety, change in mood and difficulties with cognition that occur all too commonly. Some women experience serious mental ill health for the first time ever in their mid-40s, while others with previously well-managed depression suddenly find their mental health deteriorates. It is apparent that for a significant proportion of the middle-aged female population, the menopausal transition is the ‘tipping factor’ that causes a significant change in mental health with subsequent deterioration in the quality of life. When menopause is discussed in our community, the most common symptoms described are hot flushes, cessation of periods and finishing fertility. However, these are ‘menopause-end’ symptoms and signs. Mental health symptoms are often experienced at the beginning of the menopause transition, often beginning in the early 40s, and are poorly recognised. This can lead to inadequate treatment of persistent mental ill health, with resultant loss of quality of life, and suicide in the worst situations.

## What are the main brain impacts of menopause?

During the long menopausal process (8–10 or more years), there are several key gonadal hormone shifts. The key hormones that fluctuate during menopause include oestrogen, progesterone, testosterone and the precursors to these important brain steroids (Herson and Kulkarni, 2022). It is critical to note that these hormones are potent neurosteroids and have multiple, crucial roles in the brain. This is often overlooked or misunderstood, with a common false belief that these hormones mainly impact ovaries, uterus and breast tissue. Oestrogen is intricately involved with the modulation of neurotransmitters such as serotonin, dopamine, glutamate, acetylcholine, γ-aminobutyric acid (GABA) and the opioid pathways in the brain (Barth et al., 2015) – all of which determine mood and cognition. As well, oestrogen has a role in maintaining neural circuits and synapses (Bustamante-Barrientos et al., 2021). Progesterone has a key role in modulating the GABA-ergic system – which is critical for anxiety moderation (Bitran et al., 1995). Testosterone also modulates these key neurotransmitter systems, including libido. Current neuroscientific evidence strongly underpins the understanding that all the key neurosteroid hormone levels fluctuate in the brain during menopause (Giannini et al., 2021; Herson and Kulkarni, 2022). These significant hormone fluctuations then create destabilising effects on neurotransmitter activity and brain circuitry (Giannini et al., 2021). The expression of this destabilisation varies widely between individuals so that some women experience debilitating mental health changes while others have compensatory mechanisms that enable minimal or no impact on mental health. The compensatory mechanisms include biological and social factors that can either promote mental health or create serious mental illnesses.

## Why are the mental health issues of menopause under recognised?

The increasing body of neuroscientific data and knowledge on the effects of fluctuating brain steroids such as oestrogen and resultant mental health effects should inform our understanding of menopausal anxiety and depression. Using this knowledge, it is apparent that the anxiety, depression and cognitive symptoms experienced by midlife women present as a different type of mental health condition that therefore needs a different treatment approach to standard mental health treatments. However, the lack of mainstream neurobiological knowledge has hampered the understanding and optimal management of menopausal mental health issues.

Further confounding the lack of recognition of menopausal mental ill health is the preponderance of population-wide surveys to determine the prevalence of menopausal depression. Considerable funding has been expended on this research which yields different results depending on the mental illness definitions used. This type of population survey inevitably produces averaged results that disservice the significant number of women with serious mental health issues. Clearly, there are huge numbers of women who do not experience mental ill health related to menopause, and when their survey answers are combined with the smaller number of depressed women, an average picture of good mental health in midlife women is presented. Current survey figures describe between 10% and 65% of all menopausal women surveyed experience mental ill health of varying severity (Freeman, 2015; Maki et al., 2019). The exact percentage varies but is somewhat irrelevant since we have a responsibility to understand and assist any and all women who experience significant mental ill health. Survey results should therefore not inform treatment guidelines or be used to dismiss women experiencing menopausal mental health issues.

Added to this is the lack of definitive laboratory tests to diagnose menopausal depression, coupled with confusing definitions of ’perimenopause’ tend to obfuscate the major mental health challenges that some women experience. Routine blood tests of peripheral gonadal hormone levels will not provide details of brain fluctuations of the gonadal hormones and their impact on neurochemistry. However, listening carefully to the woman experiencing mental health changes by taking a comprehensive clinical history often reveals the sudden mental health shift in her mid-40s. That should prompt consideration of the onset of the menopause transition, when other obvious causal factors for her mental ill health are lacking. A hormone treatment strategy along with other psychosocial interventions may provide the desired outcome for this woman, but it is rarely offered. More commonly, standard antidepressant medications are prescribed with only partial efficacy (Kim and Joffe, 2006).

## Optimal treatment of menopausal mental health issues

The use of menopause hormone therapy (MHT) is a much-debated topic. In particular, its use for the treatment of menopause-related mental health issues is not approved in most menopause guidelines, except for the National Institute for Health and Care Excellence (NICE, 2015) guidelines, that has a vague reference to MHT being useful in menopausal mental health. The reasons for the reluctance to include MHT in the treatment of menopausal mental ill health are based on (1) historical beliefs that mental health issues are separate to menopausal hormone shifts, (2) a lack of clinical trials’ evidence for MHT use in menopausal mental ill health conditions and (3) residual concerns over the Women’s Health Initiative (WHI) studies from 2002 (Rossouw et al., 2002). To refute these in turn: (1) The growing body of neuroscience evidence shows clear roles for gonadal hormones as potent brain steroids (Fester and Rune, 2021; McEwen and Parsons, 1982) – hence common sense dictates that MHT is a useful treatment when mental ill health is caused by menopausal hormone shifts. (2) Unfortunately, there are only a few clinical trials comparing MHT to antidepressant therapy in menopausal depression and anxiety. One reason for this is due to the prevailing erroneous view that gonadal hormone fluctuations do not cause mental ill health. This view is held by key leaders in research, healthcare and governments who do not prioritise research funding into menopausal mental health. (3) Finally, the WHI findings have been severely criticised and should not inform current MHT practice. However, the sensationalist newspaper headlines in 2002 about the WHI study claiming that hormone therapy caused breast cancer and cardiovascular disease unfortunately seem to have lingered in the memories of the general public and many clinicians too.

## The politics of menopause

Menopause is inevitable for all people assigned female at birth. Half of the world’s population experiences menopause. Inevitably, there are many different views about this biological event with its diverse psychosocial and cultural associations. In Australia, the topic of menopause has undergone generational shifts in attitude. For centuries, the approach to menopause was that it is a ‘secret women’s business’ and engendered a sense of shame in middle-aged women. This shame was often about ageing and the loss of fertility, which was equated with becoming a ‘burden’ on the community. The life expectancy of women has increased by more than 20 years compared to female life expectancy last century. The nature of women’s lives has dramatically changed in that time due to successful feminism movements. The first wave of feminism in the 1960s and onwards challenged gender roles, so that women ‘were allowed’ to participate in the workforce and at home. However, the attitude to menopause driven by the early feminists was to urge women to be quiet about menopause or any hormone-related issues such as perinatal depression or premenstrual depression – for fear that women would be seen as ‘being driven by their hormones’ and hence would not obtain senior roles in the workforce. Sadly, this attitude, although understandable in its time, is still prevalent and does not assist women to understand or discuss menopausal mental health issues and receive optimal help. It also propagates the belief that men decide what women are permitted to do in Australia. Other ideologies include the concern that in raising issues about menopausal mental ill health or hormone therapy, the natural process of menopause is being ‘pathologised’ or ‘medicalised’. This has its origins in concerning past medical practices where women received unnecessary interventions for childbirth and infant feeding, and widespread hormone replacement therapy was prescribed for healthy middle-aged women.

These views were important in the past, to enable women to take charge of their own bodies, but it is now time for a new approach to menopause that gives women greater command over their mental and physical health as well as all their diverse roles.

## A new approach for menopausal mental health

As with other mental health areas, the person with lived experience must be a key driver and collaborator in determining what treatment approaches she wants. In the area of menopausal mental health, the woman experiencing hormone-created anxiety, depression, cognitive changes (known as ‘brain fog’) and physical health issues related to menopause needs to decide what her individualised care programme includes, based on well-informed options. Many women are not aware that the menopausal process can have major impacts on mental health, which means that community education programmes need to include this information. Moreover, when framing community education and conducting assessments, it is essential to consider not only the individual’s lived experience but also their cultural background.

MHT is an important part of the treatment options available for menopausal mental health issues, particularly in the early perimenopause timeframe. The newer forms of MHT given in safer delivery modes potentially offer better outcomes for menopausal mental health and physical health issues. Coupled with comprehensive baseline and follow-up investigations, MHT may provide better resolution of menopausal depression, anxiety and ‘brain fog’ than current psychiatric medications that provide partial relief but have many side effects. Of course, healthy lifestyle advice, psychotherapy where desired and good physical health measures all have a part to play in helping women achieve their goals in their midlife years. Critically, it is the woman herself who needs to decide what the risks and benefits are for all treatments, with information from her professional treating teams. To allow this, a change is required in current MHT guidelines, so that future guidelines recommend the use of MHT in the treatment of menopausal mental health issues.

It is critical for all healthcare professionals, especially mental healthcare clinicians, to be skilled in good clinical history taking to enable early detection of menopause-related mental health issues. To assist clinicians, a validated questionnaire called MENO-D has been developed for use in any clinical practice environment (Kulkarni et al., 2018). Clinicians also need to be skilled in weighing the risks and benefits of all treatments, especially MHT in treating mental health symptoms. Professionals from many diverse backgrounds are needed to work together on new, comprehensive approaches for menopause.

It is time to innovate the approach for women with menopause-related mental health issues. To do this, we need to conduct different types of research including relevant clinical trials, to involve industry groups and corporate workplaces, to include diverse health and mental health professionals and primary healthcare practitioners and to educate all our communities about menopause using multimedia channels. The diversity of input needed for a new approach to menopausal mental health is reflected in the large, diverse group of authors listed on this viewpoint article. Above all, true innovation is only possible when the woman with lived experience of menopause is front and centre of this debate.
